# Supplementary material for: A microbiome-derived nutrient underlies tyrosine metabolism and predator avoidance in mosquito larvae
Source: bioRxiv. 2025 Apr 25:2025.04.02.646203. Originally published 2025 Apr 6. Preprint. [Version 2] doi: 10.1101/2025.04.02.646203 (PMC11996489; doi:10.1101/2025.04.02.646203)
Supplement: Supplement 1 [file media-1.pdf]

Supplemental Information

A microbiome-derived nutrient underlies tyrosine metabolism and predator avoidance in mosquito larvae

Melissa Kelley[1,*], Shubham Rathore[1], Karthikeyan Chandrasegaran[2], Cassandra Herbert[3], Chloe Elizabeth G. Sabile[1], Taylor Wood[1], Judd Joves[1], Andrew Palacios[1], Emily Susanto[1], Melissa Uhran[1], Arturo Ledezma Ramírez[1], Shyh-Chi Chen[1], Joshua Tompkin[1], Khwahish Singh[1], Muhmmad S. Khalid[1], Clement Vinauger[2], Elke Buschbeck[1], Patrick A. Limbach[3], and Joshua B. Benoit[1,*]

[1]Department of Biological Sciences, University of Cincinnati, Cincinnati, OH 45211

[2]Department of Biochemistry, Virginia Polytechnic Institute and State University, Blacksburg, VA 24061, USA

[3]Rieveschl Laboratories for Mass Spectrometry, Department of Chemistry, University of Cincinnati, Cincinnati, OH 45221

*Authors for correspondence

Melissa Kelley and Joshua B. Benoit

Department of Biological Sciences,

University of Cincinnati, Cincinnati, OH

Email: kelleym3@mail.uc.edu and joshua.benoit@uc.edu

Phone: 513-556-9714

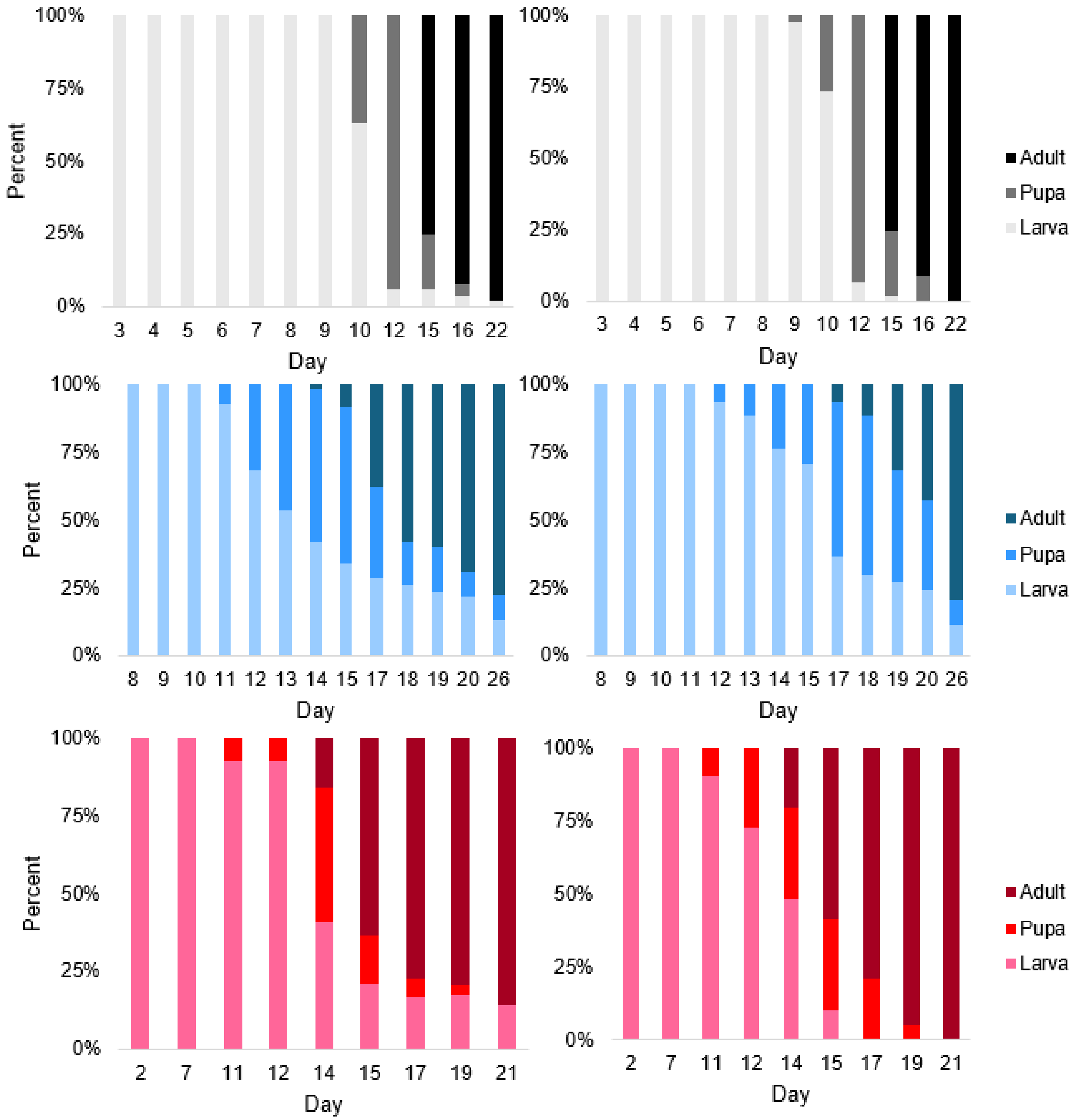

Figure S1. Transitions in developmental stage were similar in ΔqueA larvae and control K12 larvae. Three biological replicates demonstrate the time to pupation and adult emergence are aligned in the larva types.

Table S1. Relative abundance of modifications detected in mosquito larvae tRNA. The peak areas were normalized with the summation of the canonical nucleosides (C, U, G, A). A Student's t-test was used to determine significance. Only Q, manQ, and Um were significantly different between the larvae groups. Canonical nucleoside levels were the same across samples, demonstrating the amount of tRNA was the same.

Modification	Q_L1	Q_L2	Q_L3	K12_L1	K12_L2	K12_L3	t-test
C	0.11904	0.12491	0.12282	0.12166	0.12768	0.12054	0.730516
U	0.0318	0.0309	0.02989	0.03292	0.02939	0.03209	0.640027
Ψ	0.00464	0.00517	0.0051	0.00479	0.0056	0.00476	0.814576
D	0.00547	0.00652	0.00643	0.00579	0.0077	0.00575	0.721547
m^3C	0.00581	0.01176	0.01632	0.00896	0.01056	0.01344	0.930352
m^5C	0.02684	0.03054	0.03289	0.02917	0.03592	0.02888	0.6919
Cm	0.0102	0.01061	0.01035	0.01027	0.01161	0.01052	0.388944
Um	0.00057	0.00063	0.00054	0.00073	0.00071	0.00086	0.027236
m^5U	0.00281	0.0032	0.00374	0.00312	0.00369	0.00309	0.895757
Ψm	7.9E-06	1E-05	1E-05	8.5E-06	1.1E-05	8.9E-06	0.986574
A	0.57925	0.5674	0.5521	0.56128	0.56787	0.56854	0.967868
I	0.00249	0.00276	0.00299	0.00252	0.00332	0.00255	0.878456
Am	0.00495	0.00322	0.00128	0.00393	0.00153	0.00399	0.997861
m^6A	0.0102	0.01002	0.01212	0.01168	0.01239	0.00958	0.706777
m^1A	0.11165	0.1304	0.13942	0.11859	0.15264	0.11943	0.836175
m^1I	0.00253	0.00298	0.00361	0.00272	0.00341	0.00272	0.82051
G	0.26992	0.2768	0.29519	0.28414	0.27506	0.27882	0.879368
ac^4C	0.00195	0.00246	0.00283	0.00224	0.00243	0.00253	0.953222
$m^{6,6}A$	0.00446	0.0046	0.00437	0.00633	0.00436	0.00489	0.293099
m^2G	0.01305	0.01501	0.01684	0.01321	0.01715	0.01353	0.850598
Gm	0.0073	0.00771	0.00821	0.00757	0.00786	0.00758	0.815447
m^1G	0.03743	0.0422	0.04642	0.03781	0.04694	0.03864	0.831271
m^7G	0.03912	0.04877	0.05702	0.04719	0.05497	0.04655	0.839003
ncm^5U	1.8E-06	1.5E-06	1.8E-06	1.6E-06	1.8E-06	2.3E-06	0.443794
$m^2{}_2G$	0.02031	0.02424	0.02909	0.02173	0.02492	0.02295	0.644551
mcm^5U	0.00023	0.00025	0.00035	0.00025	0.00025	0.00029	0.652329
i^6A	0.00282	0.00349	0.00446	0.00309	0.00296	0.00394	0.677593
acp^3U	0.0011	0.00034	0.00023	0.00077	0.00037	0.00063	0.928232
acp^3D	0.00013	2.7E-05	1.4E-05	8E-05	4.3E-05	4E-05	0.926351
Q	2.5E-05	3.8E-05	0.00031	0.00095	0.00103	0.00138	0.003623
t^6A	2.8E-05	3.2E-05	4.5E-05	3.8E-05	4.9E-05	2.9E-05	0.668692
oQ	3.5E-07	4.3E-07	2.1E-05	1.1E-06	2.2E-06	1.6E-06	0.460256
m^6t^6A	1.3E-06	1.6E-06	9.3E-07	7.3E-07	1.4E-06	1.3E-06	0.64714
ms^2t^6A	5.2E-05	8.2E-05	7.2E-05	6.7E-05	3.8E-05	0.00016	0.642832
manQ	3.2E-05	5E-05	0.00037	0.00075	0.00084	0.00106	0.006841

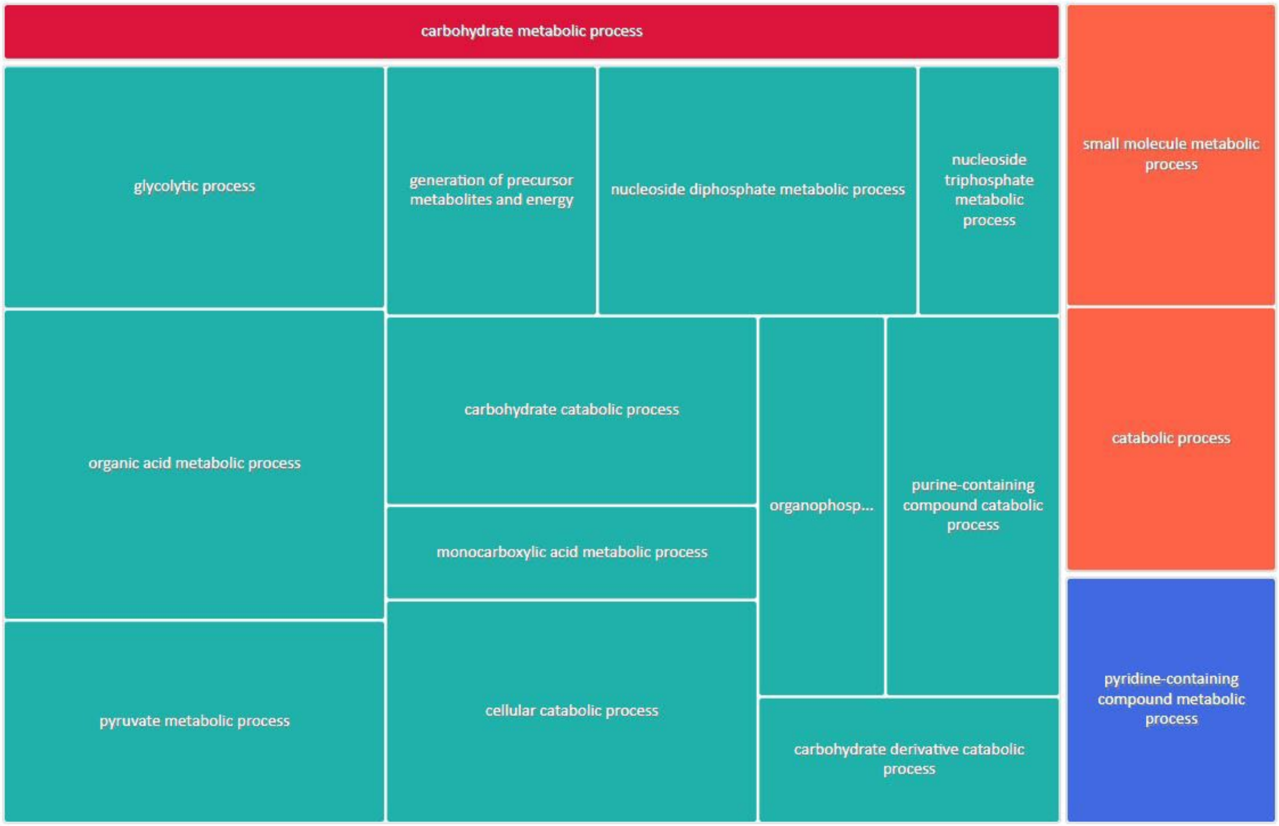

Figure S2. Transcripts that were downregulated in ΔqueA larvae compared to K12 controls such as catabolism, carbohydrate metabolism, and other metabolic processes.

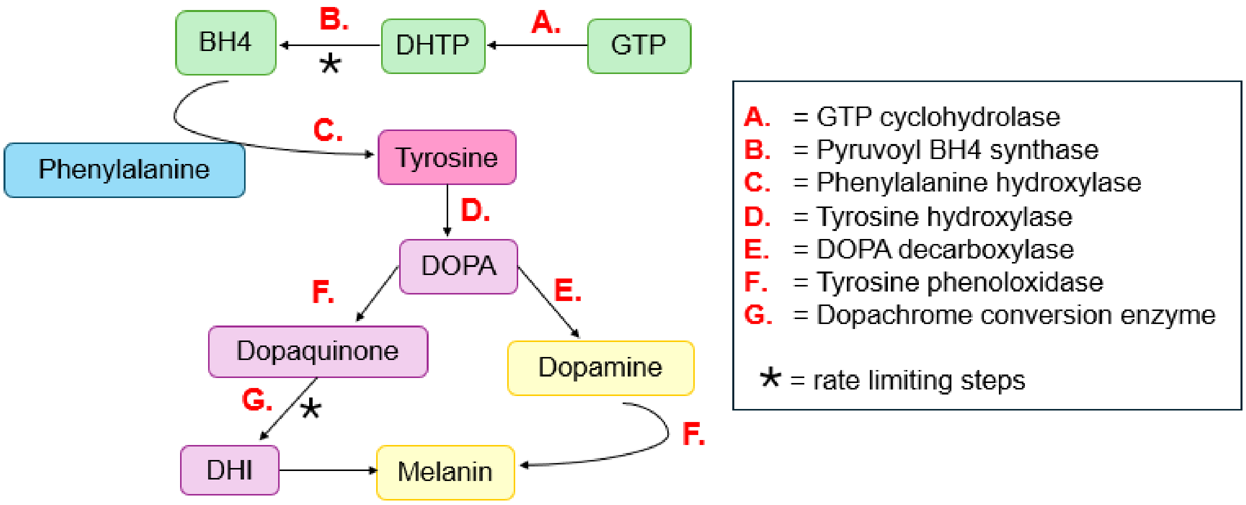

Figure S3. Pathways which rely on tyrosine as a precursor. Tyrosine is converted to L-dopa and then L-dopa can be converted to DHI or dopamine. The enzyme names and corresponding reactions are found here.

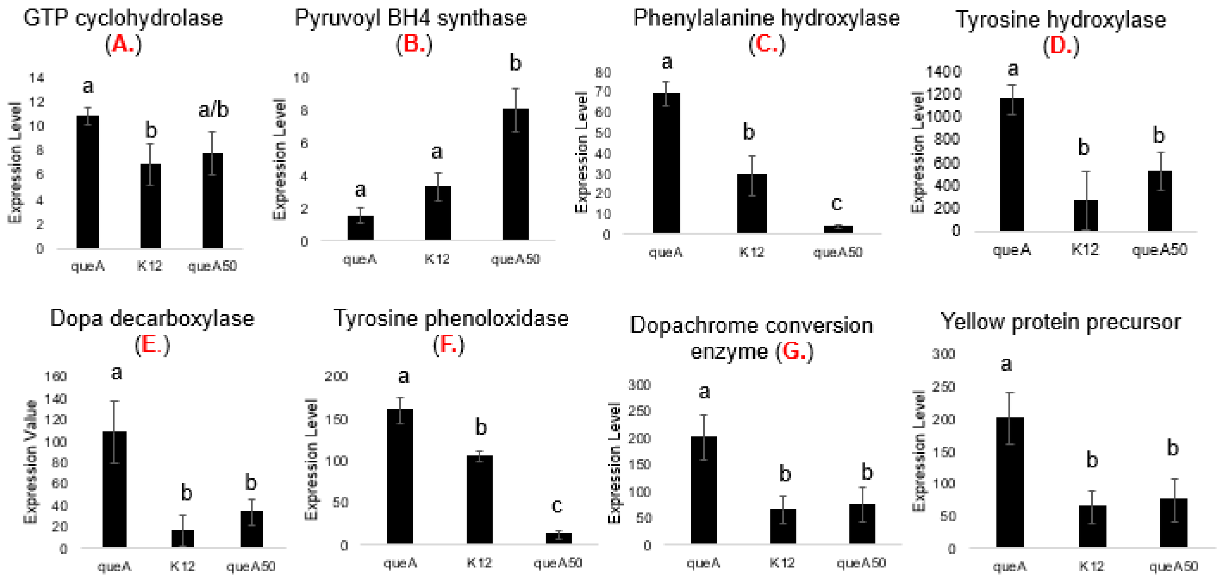

Figure S4. Expression values of specific enzymes involved in the tyrosine-dopa-dopamine pathway. Letters in red indicate pathway step shown in Figure S3. Expression values were determined significant with a p value < 0.05.

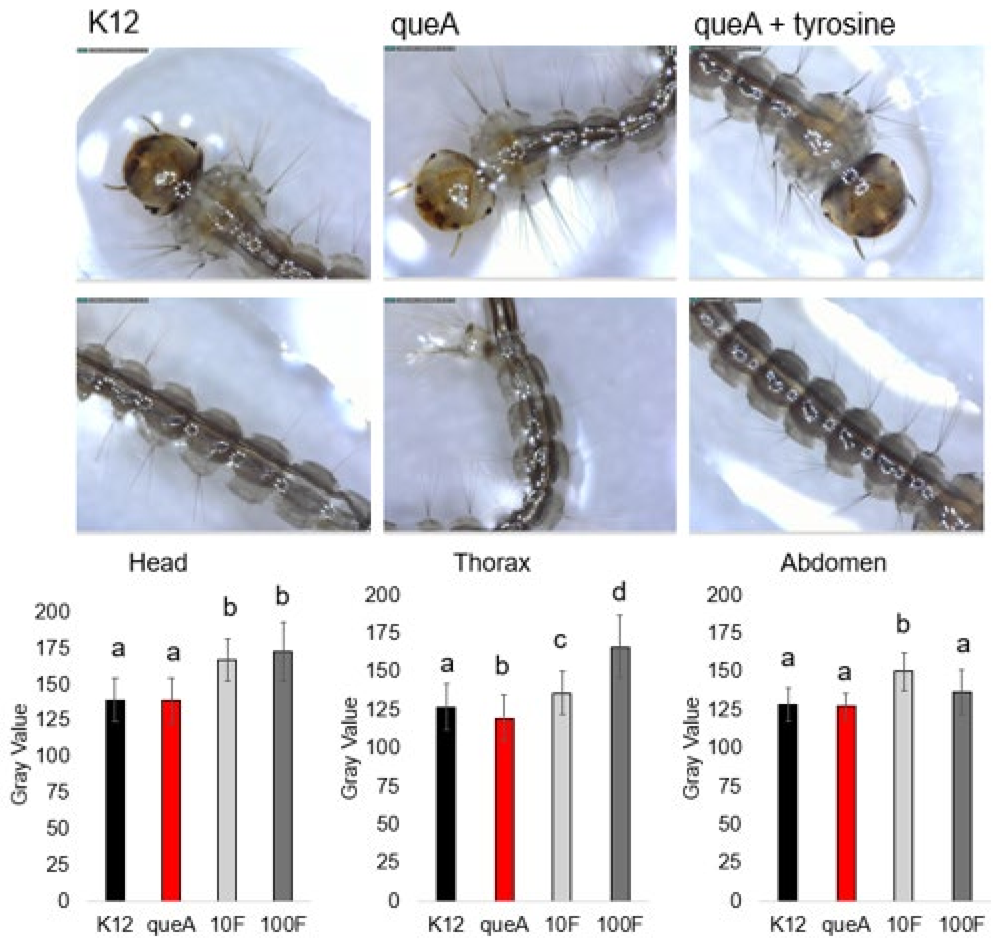

Figure S5. Tyrosine supplementation recovers the cuticular tanning defects observed in the ΔqueA larvae.

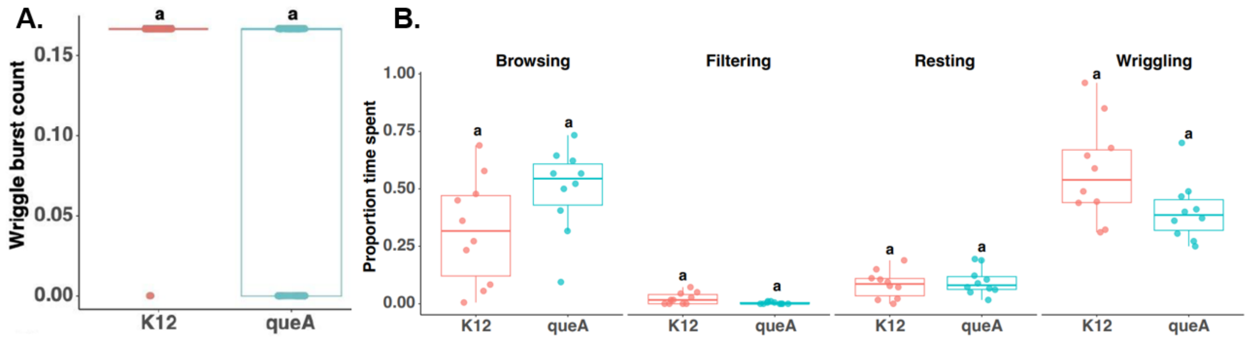

Figure S6. A. The overall wriggle burst counts were the same despite Q levels. **B.** The proportion of time spent on specific behaviors were the same regardless of Q levels in mosquito larvae.

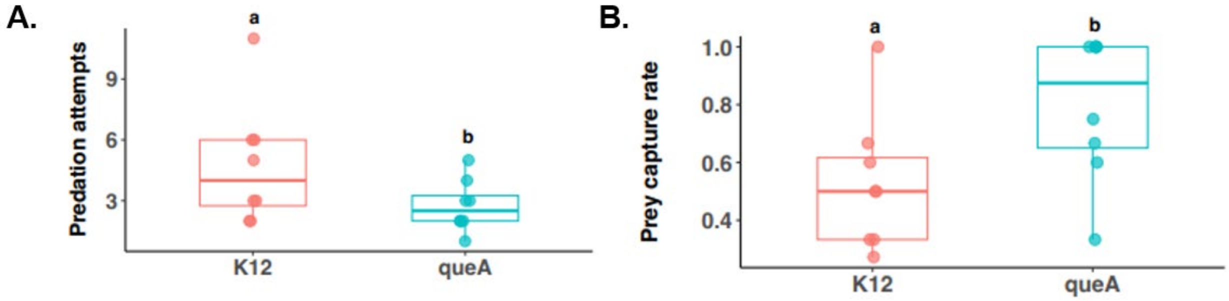

Figure S7. More predation attempts were necessary for the predator to capture K12 larvae. The prey capture rate was higher in ΔqueA larvae than K12, indicating queA larvae are captured more easily by the predator.

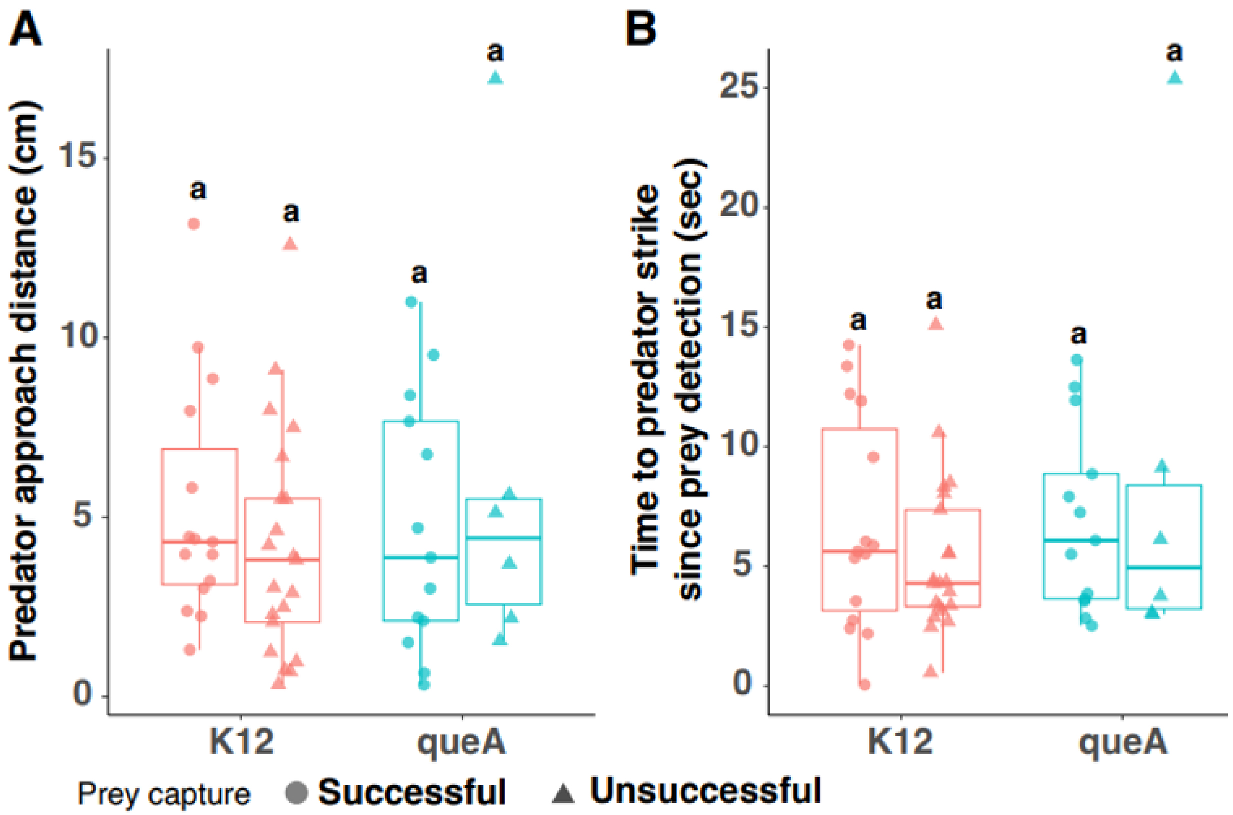

Figure S8. The predator approach distance and time to strike were similar regardless of Q levels in mosquito larvae, indicating the predator behavior is not altered during hunting.

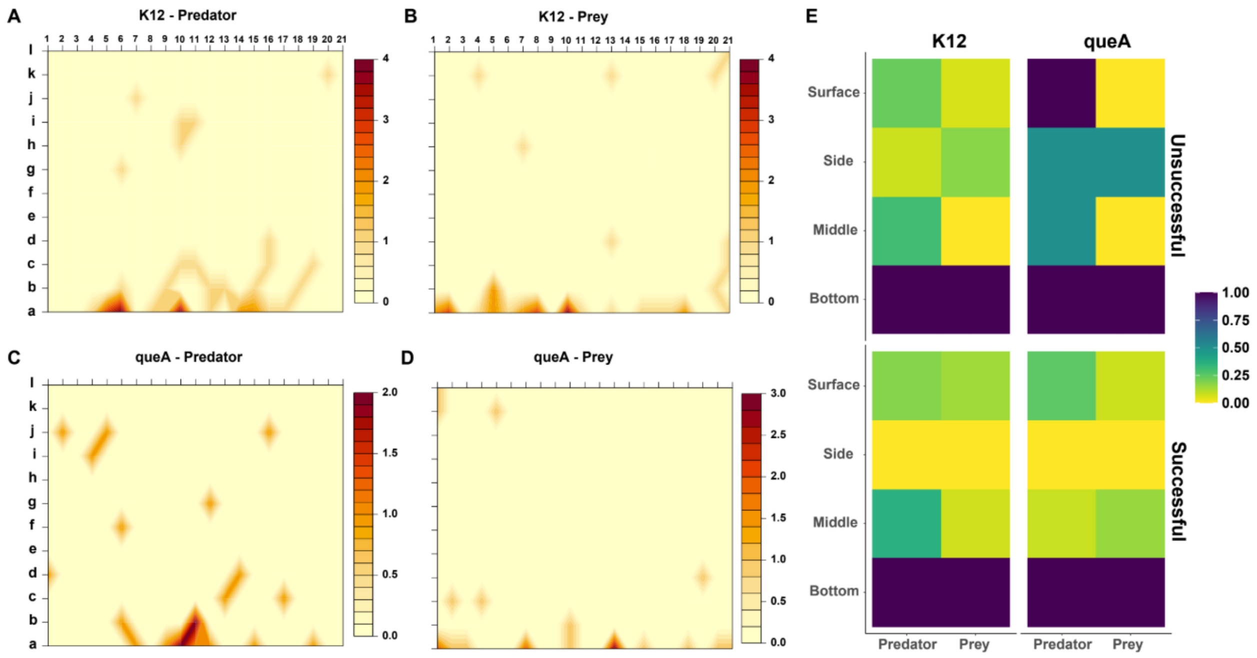

Figure S9. Predator-prey dynamics suggest that ΔqueA larvae were only found at the bottom of the vertical arena. This distinction in space use suggests that ΔqueA larvae that successfully evaded predators did so not through active escape behaviors but likely by taking advantage of non-overlapping spatial positioning with predators. ANOVA, $P < 0.05$. N = 24 per treatment.

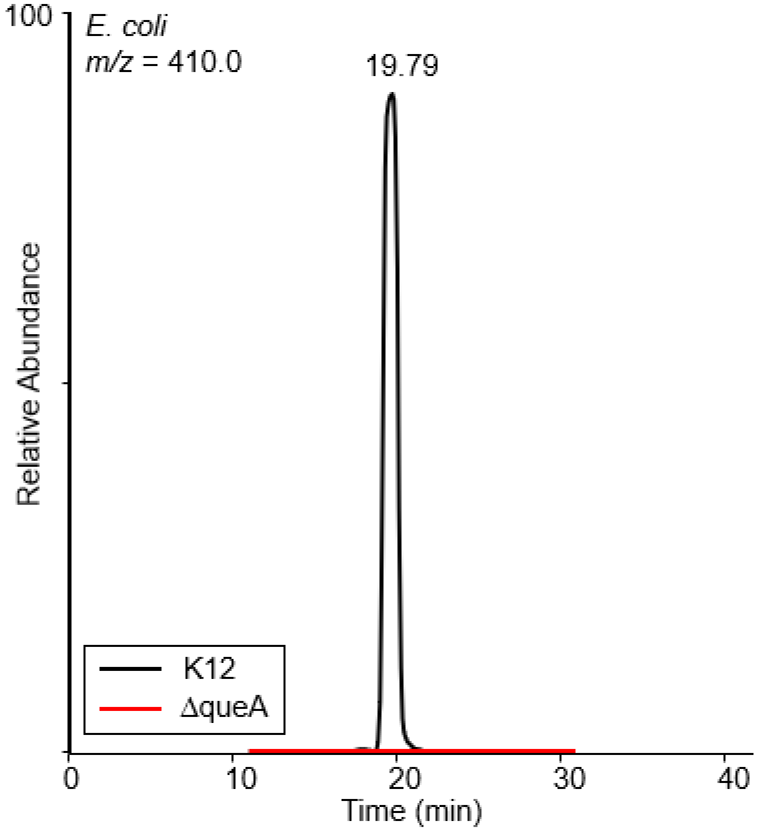

Figure S10. TIC of Q in the parental K12 *E. coli* strain (black) and the absence of Q in the ΔqueA strain(red) tRNA.

Table S2. Single reaction monitoring (SRM) transitions used for identification and quantification of nucleosides in mosquito larvae tRNA.

Compound	Retention Time (min)	RT Window (min)	Polarity	Precursor (m/z)	Product (m/z)	Collision Energy (V)	RF Lens (V)
C	2.48	3	Positive	244	112	12	41
U	4	8	Positive	245	113	10	30
Y	1.71	3	Positive	245	209	10	30
D	3	6	Positive	247	115	10	43
Cm	7.99	6	Positive	258	112	12	41
m3C	5.55	6	Positive	258	126	14	46
m5C	10	20	Positive	258	126	14	46
m1Y/m3Y	5.92	6	Positive	259	169	35	96
m5U	9.64	6	Positive	259	127	10	30
Um	14.17	6	Positive	259	113	10	30
Ym	5.72	10	Positive	259	223	10	30
A	23.3	10	Positive	268	136	17	63
I	9.23	6	Positive	269	137	10	40
m5Um	27.08	10	Positive	273	127	35	96
m5Um	27.08	10	Positive	273	241	35	96
Am	33.39	10	Positive	282	136	17	63
m1A	4	8	Positive	282	150	20	74
m6A	5.17	10	Positive	282	150	20	74
m1I	19.13	8	Positive	283	151	20	74
G	10.61	8	Positive	284	152	14	46
ac4C	22.68	6	Positive	286	154	10	46
m6,6A	33.67	8	Positive	296	164	28	79
Gm	20.43	10	Positive	298	152	11	50
m1G	23	20	Positive	298	116	16	50
m2G	30	33	Positive	298	166	16	50
m7G	8.65	10	Positive	298	166	16	50
ncm5U	5	8	Positive	302	170	35	96
m2,2G	31.2	10	Positive	312	180	15	60
mcm5U	23.39	15	Positive	317	125	10	30
mcm5U	23.39	15	Positive	317	153	10	30
mcm5U	23.39	15	Positive	317	185	10	30
m2,2,7G	31.21	10	Positive	326	194	31	96
mcm5s2U	34.06	20	Positive	333	141	35	96
mcm5s2U	34.06	20	Positive	333	169	35	96
mcm5s2U	34.06	20	Positive	333	201	35	96
i6A	36.94	6	Positive	336	136	31	96
i6A	36.94	6	Positive	336	148	31	96
i6A	36.94	6	Positive	336	204	31	96
acp3U	3.07	6	Positive	346	214	10	50
k2C	18	15	Positive	372	240	12	41
ms2i6A	41.41	8	Positive	382	182	31	96
ms2i6A	41.41	8	Positive	382	194	31	96
ms2i6A	41.41	8	Positive	382	250	31	96
tm5U	30	60	Positive	382	250	10	50
ct6A	34.81	6	Positive	395	263	31	96
ms2io6A	36.31	4	Positive	398	266	31	96
tm5s2U	36.74	15	Positive	398	266	35	96
Q	21.02	10	Positive	410	163	35	96
Q	21.02	10	Positive	410	295	35	96
t6A	31.43	10	Positive	413	281	31	96
oQ	16.5	7	Positive	426	163	29	84
oQ	16.5	7	Positive	426	295	12	84
m6t6A	32.49	4	Positive	427	295	31	96
ms2t6A	36.04	15	Positive	459	208	31	96
ms2t6A	36.04	15	Positive	459	327	31	96
galQ	25.85	10	Positive	572	163	35	96
galQ	25.85	10	Positive	572	295	35	96
manQ	22.94	10	Positive	572	163	35	96
manQ	22.94	10	Positive	572	295	35	96
ISTD	18.7	10	Positive	613	306	12	41
